# Cutaneous T-Cell Lymphoma in Asians

**DOI:** 10.5402/2012/575120

**Published:** 2012-07-15

**Authors:** Min Soo Jang, Dong Young Kang, Jong Bin Park, Sang Tae Kim, Kee Suck Suh

**Affiliations:** Department of Dermatology, Kosin University College of Medicine, 34 Amnam-Dong, Seo-gu, Busan 602-702, Republic of Korea

## Abstract

Cutaneous T-cell lymphoma describes a heterogeneous group of neoplasms of skin homing T cells that vary considerably in clinical presentation, histologic appearance, immunophenotype, and prognosis. This paper addresses the cutaneous T-cell lymphoma in Asians with respect to clinical-epidemiologic and histopathological features. Compared with Western countries, Asia usually has higher rates of cutaneous T-cell lymphomas such as extranodal NK/T-cell lymphoma, hydroa vacciniforme-like lymphoma, subcutaneous panniculitis T-cell lymphoma, and adult T-cell leukemia/lymphoma and lower rates of cutaneous B-cell lymphomas. Among many variants of mycosis fungoides, hypopigmented lesions, pityriasis lichenoides-like lesions, and ichthyosiform lesions are more prevalent in Asia than in the West. Adult T-cell leukemia/lymphoma is endemic in southwestern Japan especially in the Kyushu island. The clinicopathologic characteristics of cutaneous lymphoma vary according to geography, and this may be ascribed to genetic and environmental etiologic factors.

## 1. Introduction

Cutaneous T-cell lymphomas (CTCLs) are non-Hodgkin lymphomas characterized by a dominant skin-homing T-cell clone with differing clinical presentations, histologic features, and therapeutic considerations. The reported incidence of these cancers has risen sharply over the past 15 years, which may be due to a combination of real increases in cases and improved access to and detection by medical practitioners [1].

The Korean dermatopathology research group reviewed nationwide collection of 80 cutaneous lymphoma cases in Korea. In this study, the most frequent cutaneous lymphoma was mycosis fungoides (42.5%), followed by anaplastic large cell lymphoma (19%), NK/T-cell lymphoma (15%), subcutaneous panniculitis-like T-cell lymphoma (11%), and cutaneous B-cell lymphomas (4%) [2]. Fujita et al. [3] reviewed 106 primary cutaneous lymphoma cases from a single Japanese medical center according to the revised 2008 WHO classification: cutaneous lymphomas comprised mycosis fungoides (52%), CD30 positive T-cell lymphoproliferative disorder (16%), adult T-cell leukemia/lymphoma (6%), NK/T-cell lymphoma (4%), subcutaneous panniculitis-like T-cell lymphoma (3%), and mature B-cell neoplasms (13%). As a whole, mature T-cell and NK-cell neoplasms were frequent (87%) because of the occurrence of adult T-cell leukemia/lymphoma and extranodal NK/T-cell lymphoma, nasal type, with less frequent occurrence of mature B-cell neoplasms (13%). Therefore, compared with Western countries, Korea and Japan usually had higher rates of cutaneous NK/T-cell lymphomas such as extranodal NK/T-cell lymphoma and subcutaneous panniculitis-like T-cell lymphoma and lower rates of cutaneous B-cell lymphomas. 

The occurrence rates for various subtypes of cutaneous lymphoma in Asia are considered to be significantly distinct from those in Western countries. However, there has not been a report summarizing schematically incidence patterns of CTCL occurring in Asians. We reviewed the clinicopathologic features of CTCL groups more common in Asia.

## 2. Mycosis Fungoides

Cutaneous lymphoma represents a heterogenous group of T-, NK, and B-cell neoplasms, with mycosis fungoides (MF) being the most common subtype. The annual incidence of MF in the USA varies from 3.6 to 4.6 cases per 10^6^ of the population showing a continued and substantial rise [4, 5]. Their recently large population-based study of 3884 cases showed an incidence of 4.1 per 1,000,000 person-years [6]. The incidence in Europe is somewhat less, but proportion of MF within cutaneous lymphoma is similar to those of USA [7]. There is predilection for males (2 : 1). Any age group may be involved, but there is a higher incidence in the fourth to sixth decades. It is more common in blacks (2 : 1) and less common in Asians and Hispanic Whites [6]. Epidemiologic investigations in USA have shown similar incidence patterns of lymphomas among foreign-born and US-born Asians, supporting the role of host susceptibility in etiology [6]. In several reports performed in several Asian countries, incidence of this entity in the cutaneous lymphoma ranged from 13% to 52% displaying various pattern [2, 3, 8, 9].

Mycosis fungoides has a plethora of clinicopathological manifestations [10]. Many variants of this lymphoma differ substantially from “classical” mycosis fungoides and are therefore sometimes referred to as “atypical” forms of the disease [10]. Atypical forms of mycosis fungoides include hypopigmented, hyperpigmented, ichthyosiform, pityriasis lichenoides-like, granulomatous, folliculotropic, bullous, palmoplantar, pagetoid reticulosis, and granulomatous slack skin [10–17]. Among these variants, hypopigmented, pityriasis lichenoides-like, and ichthyosiform mycosis fungoides are more prevalent in Asians [11–15].

### 2.1. Hypopigmented Mycosis Fungoides

Hypopigmented mycosis fungoides is overwhelming in Asians, with only 16 cases have been reported so far in Caucasians [2, 14, 18, 19]. Compared to other clinical manifestation of mycosis fungoides, the hypopigmented mycosis fungoides is more prevalent in young age group [10, 14, 19].

**Figure 1:**
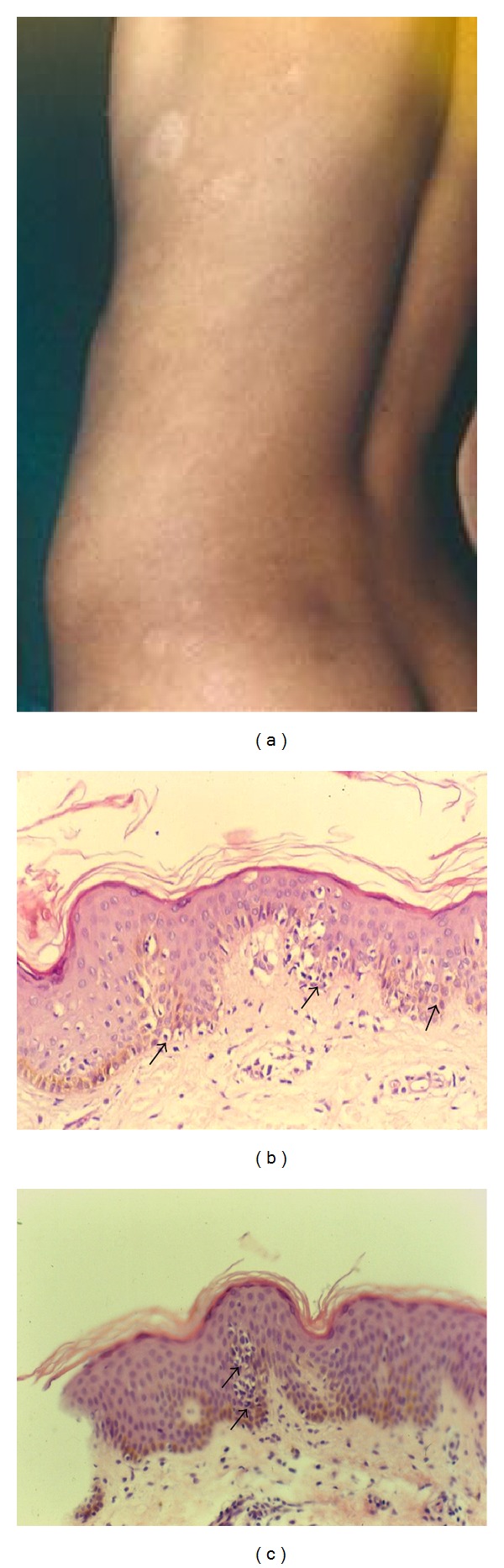
(a) Hypopigmented mycosis fungoides. Various sized, hypopigmented, scaly macules, and patches on the trunk. (b) Numerous atypical mononuclear cells (arrow) surrounded by clear halos are scattered through the epidermis are seen (H&E, ×200). (c) A collection of atypical hyperchromatic lymphocytes (arrow) without spongiosis is seen (H&E, ×400).

 The lesions may be misinterpreted clinically as those of pityriasis versicolor, pityriasis alba, vitiligo, leprosy, sarcoidosis, and postinflammatory hypopigmentation. Histologically hypopigmented mycosis fungoides lacks epidermal atrophy and demonstrated moderate to marked epidermotropism resembling pagetoid reticulosis [10, 14] (Figure 1).

Although hypopigmented mycosis fungoides may present as the sole manifestations of mycosis fungoides, in some cases, especially in Caucasians, careful examination of the patients will detect the presence of erythematous lesions as well.

Recent studies have shown that neoplastic cells in hypopigmented mycosis fungoides often express CD8. In response to treatment, perifollicular repigmentation may be seen [10, 17].

The pathogenesis of hypopigmented mycosis fungoides is still unclear. Hypomelanosis may be due to the cytotoxic effect of T suppressor lymphocytes in melanocytes. The peculiar clinical change might result from a decreased transfer of melanosome from melanocytes to keratinocytes and melanocyte degeneration as evidenced by electron microscopic studies [10, 20].

In addition, the majority of melanosomes are spherical type and incompletely melanized. Such ultrastructural changes are not specific for hypopigmented mycosis fungoides and found in a variety of acquired hypopigmentary disorders [20].

Hypopigmented mycosis fungoides is characterized by good response to therapy, particularly to PUVA, with biologically benign course, although recurrence is common [10, 12, 18].

### 2.2. Pityriasis Lichenoides-Like Mycosis Fungoides

**Figure 2:**
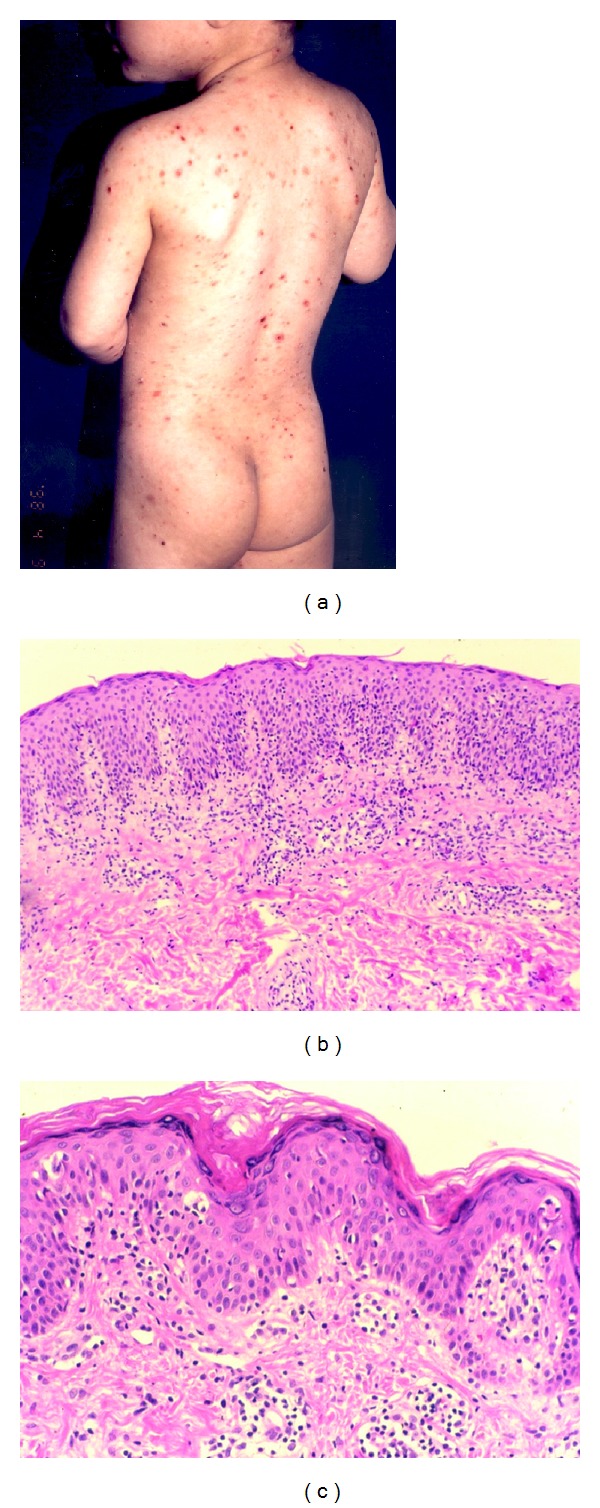
(a) Pityriasis lichenoides-like mycosis fungoides. Brown red scaling papules and macules, and some erythematous lesions with a hemorrhagic crust may be seen. (b) There is marked disproportionate epidermotropism (H&E, ×100). (c) There are slight focal epidermotropism, Pautrier's microabscess, and coarse collagen bundles (H&E, ×200).

 A peculiar variant of mycosis fungoides simulated clinically and histopathologically the picture of pityriasis lichenoides and is particularly difficult to diagnosis [15] (Figure 2). This variant of mycosis fungoides was exclusive in Asians, especially in children [15, 19, 21]. Recently, clinico-epidemiological study performed in Kuwait indicated that pityriasis lichenoides chronica-like MF accounted for 3% of MF [19]. We think this variant is mostly restricted to Asians and children.

### 2.3. Ichthyosiform Mycosis Fungoides

**Figure 3:**
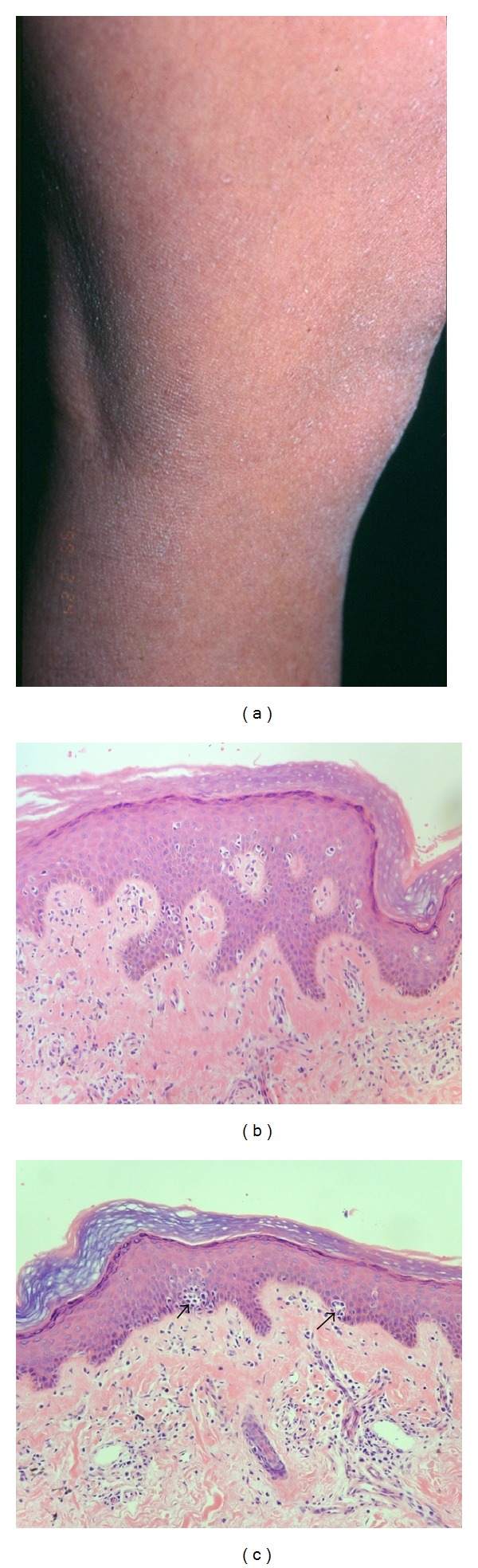
(a) Ichthyosiform mycosis fungoides. Ichthyosiform eruption located on lower extremities. (b) There are compact orthokeratosis with underlying thinned granular layer and slight focal epidermotropism (H&E, ×100). (c) Pautrier's microabscess (arrow) composed of atypical hyperchromatic lymphocytes are seen (H&E, ×100).

Ichthyosiform mycosis fungoides is a rare variant of mycosis fungoides. Although the clinical features are indistinguishable from those of acquired ichthyosis, the histopathologic findings reveal epidermotropic infiltrates composed of cerebriform lymphocytes typical for mycosis fungoides [10, 11] (Figure 3).

It represents itself clinically as widespread ichthyosiform lesions often accompanied by comedo-like lesions and/or follicular keratotic papules. These ichthyosiform lesions favor the extremities, but whole body may be involved [10]. We have observed that ichthyosiform eruption appears in conjunction with the hypopigmented lesions or angiocentric lesions in our recent study (unpublished data).

 While the ichthyotic changes are usually the only manifestations of this mycosis fungoides variant, the combination of “classical” mycosis fungoides and acquired ichthyosis (as a paraneoplastic phenomenon) have been documented [11].

Histologically, the ichthyosiform area shows compact orthokeratosis and hypogranulosis that are characteristic findings for acquired ichthyosis. In addition, band-like epidermotropic infiltrates composed of small cerebriform lymphocytes typical for mycosis fungoides [11] (Figure 3).

In recent studies, ichthyosiform mycosis fungoides represents 1.8% of mycosis fungoides cases in Italy, 3.5% in Israel, 14% in Japan, and 4.2% in Korea (unpublished data), occurring more frequently in Asia [11–13].

## 3. NK/T-Cell Lymphoma

**Figure 4:**
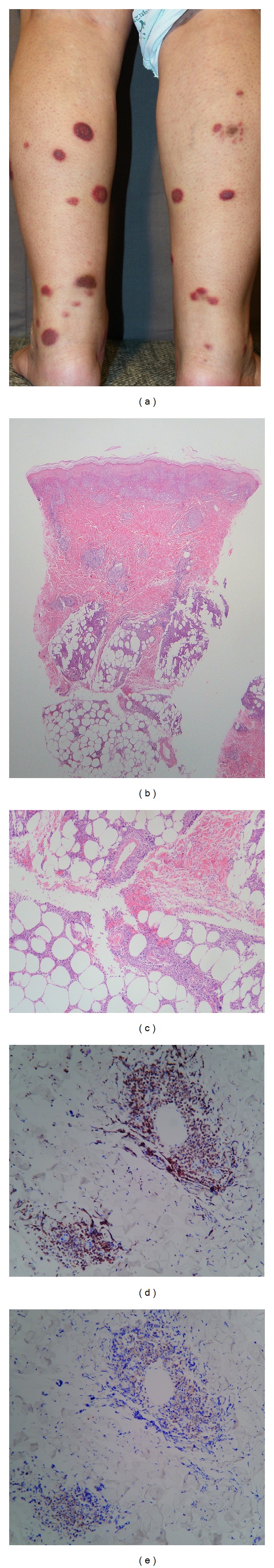
(a) Extranodal NK/T-cell lymphoma presenting as violaceous nodules on the leg. (b) Neoplastic lobular lymphoid infiltrate in the dermis and subcutis (H&E, ×20). (c) Prominent angiodestruction and extensive necrosis with diffuse infiltration of various cells (H&E, ×200). (d) Positivity of neoplastic cells for cytoplasmic CD3 and (e) CD 56.

Natural killer/T-cell lymphoma (NKTCL) is characterized by angiocentric and angiodestructive infiltrates of malignant cells with NK- or cytotoxic T-cell phenotype. Most cases are derived from NK cells, but rare cases have a cytotoxic T-cell phenotype. This rare type of lymphoma preferentially involves the upper respiratory tract, especially the nasal cavity and nasopharynx, but also shows a predilection for the skin, second most affected site, and used to be referred to as polymorphic reticuloses or angiocentric immunoproliferative lesion (Figure 4). Extranodal NKTCL is renowned for its strong association with the Epstein-Barr virus (EBV) and characteristic midline facial granuloma in the nasal cavity [18].

Extranodal NKTCL tends to more often affect adult male at a median age of 50 years. It is more common in East Asian countries such as China, Hong Kong, Korea, and Japan, rare in Europeans, and relatively frequently encountered in Native Americans in Mexico and South and Central America [22–24]. The International Peripheral T-Cell Lymphoma Project reported a fourfold higher relative frequency of extranodal NKTCL among lymphomas in Asian countries compared to Western countries. It is relatively common in Korea, with a marked predominance of the NK-cell phenotype, comprising up to 10% of all non-Hodgkin's and more than 70% of lymphomas arising in the nasal cavity and paranasal sinuses. Furthermore, extranodal NKTCL presenting with cutaneous manifestation primarily compromised 20% of a total of 100 cases of primary cutaneous lymphoma in a study performed in Korea between 1998 and 2001 [2, 25]. In Taiwan, it is reported to be the most common form of CTCL [9].

The different incidence of EBV-related disorder appears to result from the ethnic susceptibility to EBV infection-associated HLA determinants or the geographical distribution of EBV infection, which may be directly associated in oncogenesis. EBV can be classified based on the dissimilarity in the sequence of 2 regions in the EBV nuclear antigen (EBNA), type A and type B. Almost all of the NKTCL cases in Asia, including Korea, Japan, and Malaysia, had type A EBV, suggesting the preponderance of type A EBV in Asia. On the contrary, some other studies in Western societies indicated type B EBV in the NKTCL [25, 26]. It seems to suggest a geographic difference in the distribution of EBV subtype in NKTCL.

## 4. Hydroa Vacciniforme-Like Lymphoma

Hydroa vacciniforme-like lymphoma (HVL) is a rare type of EBV-associated lymphoma of cytotoxic T-cell or NK-cell origin that mainly affect children, characterized by a vesicopapular skin eruption that clinically resemble hydroa vacciniforme (HV). It was considered by the WHO-EORTC to be a new variant of extranodal NKTCL, nasal type. However, in the revised version of the 2008 WHO of lymphoma, the disease is introduced and regarded as the new entity, reflecting chronic course and occasionally spontaneous involution contrary to the previous 2005 WHO-EORTC classification [27].

The annual incidence of HVL is not accurately estimated in Asians due to fact that this entity is newly listed in the WHO classification of 2008 and difficult to differentiate from HV. It is predominantly reported in Latin America (Peru, Mexico, and Guatemala) and Asia (Korea, Japan, and Taiwan), and rarely, it occurs in Caucasians [18, 25, 28].

This condition have been also described as edematous scarring vasculitic panniculitis and angiocentric CTCL of childhood [29, 30]. Cases referred to as severe hydroa vacciniforme-like eruption are probably part of the spectrum. Skin lesion is characterized by an edematous crusting papulovesicular eruption usually on light-exposed skin, mainly the face and upper limbs. The disease is exacerbated in the summer season and may wane during the winter months. Unlike typical HV, cutaneous lesions may also occur on nonexposed sites and minimal erythema dose phototesting is nonspecific. Lesions show edema, papules, and blisters and progress to ulceration and varicelliform scars. Systemic symptoms such as fever, hypersensitivity to mosquito, lymphadenopathy, hepatosplenomegaly anemia, and leukopenia may develop, particularly late in the course of the disease. Hypersensitivity to mosquito bites (HMBs) shows exaggerated reactions to mosquito bites including necrotic skin eruption and various general symptoms (high fever, general malaise, cramps, bloody stools, wheezing, hematuria, and proteinuria). It is very uncommon, and cases were reported in mostly in East Asia, including Japan, Korea, and Taiwan and Latin America, such as Mexico, interestingly identical to countries where HVL is prevalent [25].

## 5. Subcutaneous Panniculitis Like T-Cell Lymphoma

**Figure 5:**
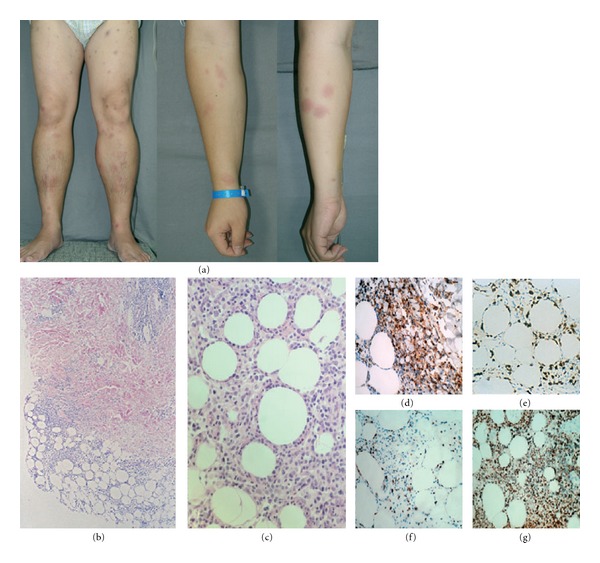
(a) Multiple tender purplish indurated patches on the both forearm and lower extremities. (b) A dense lymphoid infiltrate located in the subcutaneous tissue (H&E, ×40). (c) A focal rimming of adipocytes by atypical lymphocytes, karyorrhexis, and phagocytic macrophages in the subcutaneous tissue (H&E, ×100). ((d), (e), (f), (g)) Immunohistochemical staining showed (d) CD3 (+), (e) CD8 (+), (f) *β*F1 (+), and (g) T-cell intracellular antigen-1 (+) (H&E, ×200).

Subcutaneous panniculitis-like T-cell lymphoma (SPTCL) is defined as a rare lymphoma that primarily infiltrates subcutaneous tissue, shows high-grade cytologic features, and is composed of CD8+, *α*/*β*+ T cells with a cytotoxic phenotype (Figure 5). The current EORTC/WHO classification therefore reserves the term SPTCL for alpha-beta positive cases, while gamma-delta cases are regarded as a separate, provisional entity, “gamma-delta cutaneous T-cell lymphoma”, within the peripheral T-cell lymphoma, unspecified category [27, 30].

According to studies by Criscone et al. and Bradford et al., SPTCL comprised under 1% of the cutaneous lymphoma [1, 6]. In addition, several reports in Europe also indicated that incidence rate of SPTCL reached almost zero [7, 31]. Studies by Fujita et al. [3], Liao et al. [9], and Lee et al. [2] enrolling Asians reported that incidence represented 2.3%, 3.0%, and 11% of all cutaneous lymphoma, respectively. Therefore, compared with the Western contries, there was a higher frequency of SPTCL in Asian nations. Notably, among the Asians, compared with other Far Eastern countries, Korea had considerably higher rate of subcutaneous panniculitis-like T-cell lymphoma [2]. However, these data were collected prior to the new WHO-EORTC classification, and thus the true incidence of SPTCL is speculated to be less than previously thought [32].

## 6. Adult T-Cell Leukemia/Lymphoma

Adult T-cell leukemia/lymphoma (ATLL) is a peripheral T-cell leukemia—lymphoma caused by the human retrovirus human T-lymphotropic virus-1 (HTLV-1). Cutaneous manifestations and histopathologic features are often identical to those of mycosis fungoides, so demonstration of retroviral infection is compulsory for the diagnosis [18]. It commonly occurs in certain endemic areas of HTLV-1 infection, including Japan, the West Indies, Central Africa, and the Caribbean. Occasionally, cases are also diagnosed in the rest of the United States and Europe as a consequence of immigration from endemic areas, especially the West Indies and Africa [33]. The distribution of infected patients is not uniform in endemic countries. Particularly, southwestern Japan, represented by Kyushu and Okinawa, and northeastern Brazil are more prevalent regions in ATLL [33]. Of all malignant lymphomas, the incidence of ATLL out of NK/T-cell neoplasms was reported to be 59% in Kyusyu and 54% in Okinawa [23]. It is intriguing to note that extremely low incidences of HTLV-1 seropositivity and ATLL were found in Korea and Eastern China, neighboring countries of Japan. Seroepidemiologic study in Korea by Lee et al. [34] represented a lower seropositivity rate for HTLV-1 (0.25%), but this study revealed an existence of HTLV-1 infection and ascertained a possible occurrence of ATLL in this area. To the best of our knowledge, occurrence of ATLL in Korea is only restricted to case representations (7 cases) [35].
